# Comprehensive analysis of hazard of ultraviolet radiation emitted during arc welding of cast iron

**DOI:** 10.1002/1348-9585.12091

**Published:** 2019-12-03

**Authors:** Jyunya Takahashi, Hitoshi Nakashima, Nobuyuki Fujii, Tsutomu Okuno

**Affiliations:** ^1^ Polytechnic University of Japan Tokyo Japan; ^2^ Graduate School of Systems Design Tokyo Metropolitan University Tokyo Japan

**Keywords:** arc welding, cast iron, effective irradiance, hazard, ultraviolet radiation

## Abstract

**Objectives:**

Ultraviolet radiation (UVR) emitted during arc welding frequently causes keratoconjunctivitis and skin erythema. The extent of the hazard of UVR varies depending on the welding process and conditions. Therefore, it is important to identify the levels of UVR present under different conditions. The purpose of this study was to investigate the degree of hazard of UVR emitted by the different types of arc welding of cast iron frequently used in industry.

**Methods:**

In this study, we experimentally measured the UVR emitted during gas tungsten arc welding (GTAW), shielded metal arc welding (SMAW), and gas metal arc welding (GMAW) of cast iron. The degree of hazard of UVR was quantitatively evaluated in accordance with the guidelines of the American Conference of Governmental Industrial Hygienists.

**Results:**

Effective irradiances measured in this study were in the range 0.045‐2.2 mW/cm^2^ at a distance of 500 mm from the welding arc. The maximum allowable exposure times corresponding to these levels were only 1.4‐67 s/day.

**Conclusions:**

UVR emitted during arc welding of cast iron has the following characteristics: (a) It is more hazardous at higher welding currents. (b) The magnitude of the hazard, which depends on the welding process, increases in the order of GMAW > SMAW > GTAW. (c) It is influenced by the filler material used; that is, the components contained in the filler material affect the hazard of UVR. The effect is Fe > Ni, Cr

## 1 INTRODUCTION

The light emitted during arc welding contains strong ultraviolet radiation (UVR). In the absence of a barrier, this radiation is emitted into the surrounding environment, resulting in extremely large numbers of workers at workplaces where arc welding is performed being exposed to UVR. This includes not only expert arc‐welding professionals—whose numbers are estimated at some 350 000 in Japan—but also welders who only perform it occasionally, as well as adjacent workers engaged in other tasks.^1^ UVR consists of electromagnetic waves with wavelengths ranging from approximately 1‐400 nm.^2^ However, the wavelength demarcating UVR from visible light cannot be precisely defined, because visual sensations at wavelengths shorter than 400 nm are noted for very bright sources. The borders necessarily vary with the application.^3^ Although UVR is not visible to the human eye, its physical properties are similar to those of visible light. The International Commission on Illumination has subdivided UVR into three wavelength regimes: UV‐A (wavelengths in the range of 315‐400 nm), UV‐B (280‐315 nm), and UV‐C (100‐280 nm).^3^ Regarding the interaction of UVR with the human eye, UV‐C is absorbed by the cornea and does not reach the interior of the eye. UV‐B and UV‐A are absorbed mostly by the cornea and the lens, and only trace amounts (<1%) reach the retina. The portion of the UV spectrum consisting of wavelengths below approximately 190 nm is known as vacuum UVR because this radiation is strongly absorbed by oxygen molecules and is not transmitted through air. Since humans are, thus, not exposed to vacuum UVR—except in extremely rare circumstances—it is not considered a hazard.

Ultraviolet radiation interacts strongly with living organisms and is known to cause a variety of problems.^4^, ^5^ Moreover, since UVR is strongly absorbed by proteins and water, when UVR is incident on a living organism, the majority of the radiation is absorbed at the surface. Thus, damage to living organisms due to UVR is confined to surface regions. Well‐known examples of its acute health effects include keratoconjunctivitis and erythema, whereas delayed health effects include cataracts and skin cancer. In practice, acute health effects due to UVR occur frequently at workplaces where arc welding is performed.^1^, ^6^ The Japan Welding Engineering Society surveyed incidences of UV keratoconjunctivitis among workers at workplaces involving arc welding—including both workers who did and did not perform arc welding.^1^ The results of the survey indicated that as many as 86% of workers experience UV keratoconjunctivitis, whereas 45% reported ongoing flare‐ups, with one or more recurrences per month. Additionally, in a survey conducted by Emmett et al,^6^ 92% of welders were found to have suffered one or more flash burns (keratoconjunctivitis), and 40% were afflicted with erythema in the neck. Moreover, the majority of arc welders experienced UV keratoconjunctivitis despite wearing welding face shields. Possible causes for this include (a) failure to put on their face shields before striking the arc, leading to exposure to UVR, and (b) exposure to UVR when adjacent workers perform arc welding at the same workplace. These findings demonstrate the need to introduce protective measures at workplaces involving arc welding in order to protect workers from UVR. This would require quantitative understanding of the hazard of UVR emitted during arc welding.

Arc welding, which is mostly applied to mild steel, is also used for welding of other metals, such as stainless steel and aluminum alloys. Among them, cast iron, which is often used in machine parts, is produced to the extent of more than 3 million tons annually.^7^ This is almost the same as the production volume of aluminum alloy products produced domestically.^7^ Cast iron is the main material used for casting, which is a process in which molten metal is poured into molds of various shapes. Since this process is relatively easy, it is suitable for mass production of large products of complex shapes. Therefore, cast iron products are used in various fields, mainly for automotive and various industrial machinery parts. Arc welding is applied to the joining of these cast iron parts and their repair.

Arc welding involves generation of an electric arc between a metal electrode and a base material (metal to be welded), with the heat generated melting and bonding the base material. It is widely and generally used as a method of joining metal materials. Among the various types of arc welding, gas tungsten arc welding (GTAW), shielded metal arc welding (SMAW), and gas metal arc welding (GMAW) are mainly used for arc welding of cast iron components.^8^, ^9^, ^10^

Gas tungsten arc welding is a welding process that uses non‐consumable tungsten electrodes. Here, a filler rod is inserted into the molten pool and an inert gas is used as a shielding gas. Since the welding electrode does not melt, the stability of the arc is excellent, and it can be applied to most metals. Additionally, the cleanliness of the weld is higher than other welding methods.

Shielded metal arc welding is a welding process involving a consumable electrode that uses a covered electrode with a coating flux around a round metal rod with a diameter of 3.2‐5.0 mm as the electrode. The main components of the coating flux are calcium carbonate and calcium fluoride.^11^ During welding, they melt together with the coated electrode to form a shielding gas to protect the molten metal and slag to remove impurities from the molten metal. Since this welding process uses relatively simple welding apparatus, it can be used in various working environments both indoors and outdoors.

Gas metal arc welding is a welding process that supplies coiled electrodes (welding wire) to welds automatically while flowing shielding gas to protect the welds. This welding process is widely used because welding wire is continuously supplied to the welding part, and various metal materials can be welded with high efficiency.

Several previous studies have measured UVR emitted during arc welding of mild steel, aluminum alloys, and magnesium alloys and assessed the hazards with respect to their acute health effects.^5^, ^12^, ^13^, ^14^, ^15^, ^16^, ^17^ The studies clarified that the hazard of UVR emitted during arc welding depends on various conditions, such as welding conditions, welding process, and welding materials. Arc welding at actual workplaces occurs under a variety of welding conditions, and the situations in which workers are exposed to the resultant UVR are highly varied as well. In recognition of these practical realities, it is important to investigate the hazards of UVRs emitted by arc welding of cast iron under a wide range of conditions, since these have not been investigated so far.

Hence, we measured UVR emitted during GTAW, SMAW, and GMAW of cast iron frequently used in industry and quantitatively evaluated their acute health hazards in accordance with the American Conference of Governmental Industrial Hygienists (ACGIH^®^) guidelines.^18^

## 2 METHODS

### 2.1 Hazard assessment of UVR

According to ACGIH guidelines,^18^ the degree of hazard of UVR, which includes various wavelengths like arc light, as a cause of acute health effects is evaluated by the effective irradiance. Effective irradiance is defined by Equation (1):

(1) $$E_{\text{eff}}=\sum_{180}^{400}E_{\lambda }\cdot S\left(\lambda \right)\cdot \Delta \lambda$$

**Figure 1:**
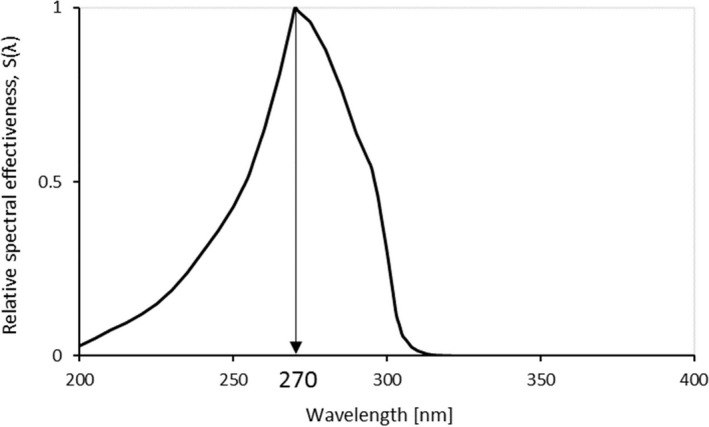
Relative spectral effectiveness.^18^ The relative spectral effectiveness indicates the degree of hazard at each wavelength and has a maximum at a wavelength of 270 nm

In this equation, *E*_eff_ is the effective irradiance (in W/cm^2^), *E_λ_* is the spectral irradiance at wavelength *λ* (in W/(cm^2^·nm)), *S*(*λ*) is the relative spectral effectiveness^18^ at wavelength *λ*, and Δ*λ* is the wavelength bandwidth (in nm). Figure 1 shows the relative spectral effectiveness,^18^ which indicates the degree of hazard present at each wavelength and has a maximum at a wavelength of 270 nm.

**Figure 2:**
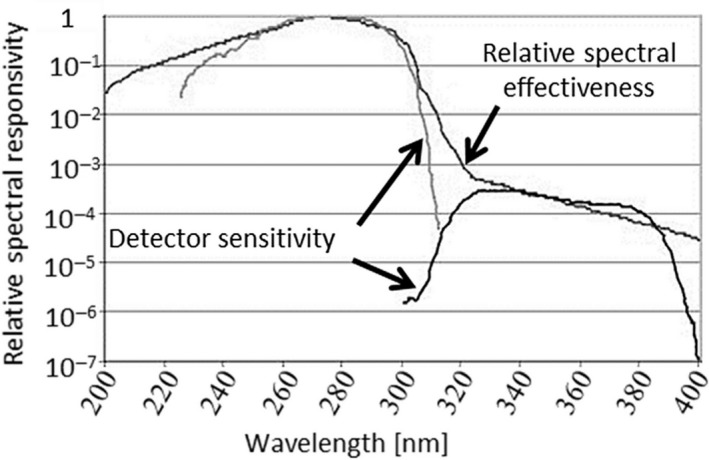
Relationship between spectral responsiveness of the UVR meter and the ACGIH relative spectral effectiveness. UVR, ultraviolet radiation

For measurements of UVR, we used an X1_3_ Hazard Light Meter and an XD‐45‐HUV UV‐Hazard Detector Head (both from Gigahertz‐Optik Inc.). These apparatuses are designed to measure effective irradiance. As shown in Figure 2, the relative spectral responsiveness of the detector head agrees well with the relative spectral effectiveness around 270 nm.^19^ Some discrepancy between the relative spectral responsiveness and the relative spectral effectiveness is visible from 310 to 320 nm; however, because the relative spectral effectiveness at this wavelength regime is small (0.015‐0.0010), we expect the impact of this discrepancy to be small and believe they cause no difficulties in practice. Thus, we concluded that this detector head is well‐suited to measurements of effective irradiance. In actual experiments, the measured value displayed by the apparatus is the effective radiant exposure (in J/m^2^). Dividing this value by the measurement time yields the effective irradiance. The measurement apparatus was calibrated by the manufacturer and was used within the one‐year interval of validity of this calibration.

**Figure 3:**
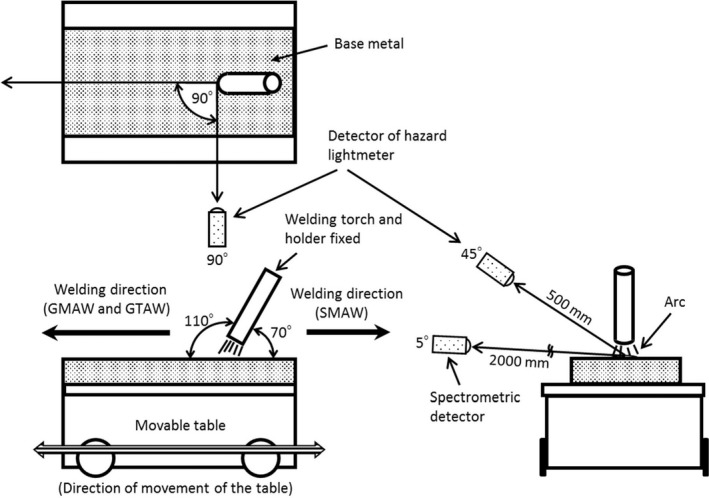
Experimental setup for measuring effective irradiance and spectral irradiance (schematic diagram)

Figure 3 shows the experimental setup for measuring effective irradiance. The position of the welding torch and holder were fixed to produce an arc in the same position, and the base metal was affixed to a movable table, allowing direct motion to enable welding. The distance between the arc and the detector head was set at 500 mm to mimic actual distance to welders. In addition, the detector head was positioned at an angle of 45° from the surface of the base metal and at an angle of 90° from the welding direction. The measurement time was set at 20 seconds. To exclude the time required for the arc to stabilize after the start of welding and the time required for the movable table to accelerate to the preset speed, measurements did not begin until 5 seconds after the start of welding. The measurement was repeated five times under each condition, and the values were averaged. In this study, no local exhaust ventilation system was used during measurement of UVR because local exhaust ventilation is usually not used in welding workplaces since it might disturb the airflow around the arc, causing welding defects.

Furthermore, following ACGIH guidelines, we divided 3 mJ/cm^2^ by our measured values of effective irradiance to determine the maximum daily exposure time allowable at that irradiance (Equation [2]).

(2) $$t_{\max}=\frac{3\;\text{mJ}/{\text{cm}}^{2}}{E_{\text{eff}}}$$

In this equation, *t*_max_ is the maximum daily exposure time (in seconds) and *E*_eff_ is the effective irradiance (in W/cm^2^).

### 2.2 Measurement of spectral irradiance

When UVR of various wavelengths is present in the arc light, it is possible to identify the element that affects the effective irradiance by measuring the intensity distribution for each wavelength. In this study, the spectral irradiance of UVR was measured to examine the influence of the components contained in the filler material on the hazard presented by UVR. Luminous elements were identified using the National Institute of Standards and Technology database.^20^

The measurement apparatus was a multichannel spectrometer (HSU‐100S, Asahi Spectra Co., Ltd.). The wavelength precision of the apparatus was ±1.2 nm. The distance from the arc was set to 2000 mm, and the measurement time was automatically set by the automatic adjustment function of the measurement apparatus. Figure 3 shows a schematic of the experimental setup for measuring spectral irradiance.

As the base metal, a spheroidal graphite cast iron plate FCD450‐10 described by the JIS^21^ was used, with dimensions of 10 × 150 × 50 mm. Table 1 shows the chemical composition of the base metal used in this study.

**Table 1:** Chemical compositions of base metal and filler materials

Material	Element (mass%)										
	C	Si	Mn	P	S	Ni	Cr	Mo	Cu	Mg	Fe
Base metal											
FCD 450‐10	3.40	2.98	0.20	0.030	0.010	—	—	—	—	0.040	Re.
Filler material											
NiFe‐1^a^	≤2.0	≤4.0	≤2.5	≤0.03	≤0.03	45‐75	—	—	≤4.0	—	Re.
ENi‐Cl^b^	≤2.0	≤4.0	≤2.5	—	≤0.04	≥85	—	—	≤2.5	—	≤8.0
ENiFe‐Cl^b^	≤2.0	≤4.0	≤2.5	—	≤0.04	40‐60	—	—	≤2.5	—	Re.
E4916^b^	≤0.15	≤0.75	≤1.60	≤0.035	≤0.035	≤ 0.30	≤0.20	≤0.30	—	—	Re.
YGW12^c^	0.02‐0.15	0.50‐1.00	1.25‐2.00	≤0.030	≤0.030	—	—	—	≤0.50	—	Re.
YS308^c^	≤0.08	≤0.65	≤1.0‐2.5	≤0.03	≤0.03	9.0‐11.0	19.5‐22.0	≤0.75	≤0.75	—	Re.

Abbreviations: GMAW, gas metal arc welding; GTAW, gas tungsten arc welding; Re., remainder; SMAW, shielded metal arc welding.

a GTAW.

b SMAW.

c GMAW.

### 2.3 Welding overview

In this study, we measured UVR emitted during the three types of arc welding (GTAW, SMAW, and GMAW) most commonly used for arc welding of cast iron.

The welding apparatus of GTAW was a digital inverter type AC/DC pulse TIG welding machine (DA300P, DAIHEN Corporation). As shown in Figure 3, the inclination of the welding torch was fixed at 110°. Using flat position forehand welding (in which the welding direction is the direction of the angle of inclination of the welding torch (110°)), bead‐on‐plate welding (in which the base metal is melted while a filler material is supplied) was performed.

The filler rod was NiFe‐1 with a diameter of 2.6 mm, as specified by JIS.^22^ The filler rod consists of 45%‐75% nickel (Ni) and about 25%‐55% iron (Fe). The shielding gas was 100% argon (Ar), and its flow rate was 10 L/min. The electrode used was a tungsten electrode (YWCe‐2), as defined by JIS.^23^ The diameter of the electrode was 1.6 or 2.4 mm, depending on the strength of the welding current. The electrode extension length was 3 mm, and the distance between the base metal and the tip of the electrode was 4 mm. The welding speed was 150 mm/min.

The welding apparatus of SMAW was an AC arc welding machine (YK‐250AD2, Panasonic Corp). As shown in Figure 3, the inclination angle of the welding holder was fixed at 70°. Using flat position backhand welding, bead‐on‐plate welding was performed (Figure 3). Only covered electrode feeding was performed manually by the welder.

The filler materials were three types of covered electrodes (ENi‐CI, ENiFe‐CI, and E4916), as defined by JIS. ENi‐CI^19^ is a covered electrode mainly composed of Ni, and ENiFe‐CI^19^ is mainly composed of Ni (40%‐60%) and Fe (40%‐60%). E4916^24^ is a low hydrogen covered electrode for mild steel that is mainly composed of Fe. The diameters of the covered electrodes were 3.2, 4.0, and 5.0 mm, depending on the strength of the welding current. The welding speed was 150 mm/min.

The welding apparatus of GMAW is a digital inverter type pulse MAG welding machine (DP350, DAIHEN Corporation). The inclination of the welding torch was fixed at 110°. Using flat position forehand welding (welding direction 110°), bead‐on‐plate welding was performed (Figure 3).

The filler material consisted of two types of solid wire (YGW12 and YS308), as defined by JIS. The main components of YGW 12^25^ and YS 308^26^ are Fe, but YS308 contains about 10% Ni and about 20% chromium (Cr). The diameter of both wires was 1.2 mm. The shielding gases were 100% CO_2_ and 98% Ar + 2% O_2_. The combination of wire and shield gas was YGW12%‐100% CO_2_ and YS308%‐98% Ar + 2% O_2_, and the flow rate of shield gases was 15‐20 L/min. The distance between the base metal and the tip of the contact tube was 17 mm, and the wire extension before the start of welding was 12 mm. The welding voltage corresponded to the welding current determined by the manufacturer of the welding apparatus. However, welding voltage was finely adjusted in consideration of the stability of the arc and appearance of the bead. The welding speed was 300 mm/min.

Table 1 shows the types of filler materials used in this study and the chemical components specified by JIS.

The welding current was of two kinds, 100 and 150 A, under all conditions. In general, in arc welding of cast iron, cracking is likely to occur due to the formation of a very hard embrittled structure in and around the base metal fusion zone.^27^ Therefore, a lower welding current during arc welding of cast iron is recommended to minimize the amount of melting of the base metal and to make the embrittlement zone as narrow as possible.

## 3 RESULTS

**Figure 4:**
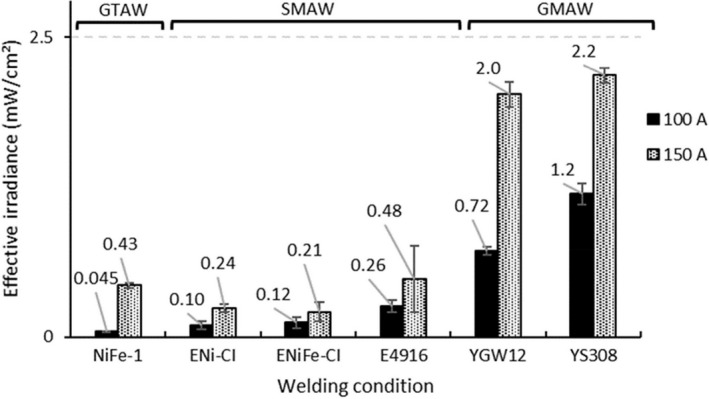
Effective irradiance of UVR emitted by arc welding of cast iron. The error bar represents the standard deviation. UVR, ultraviolet radiation

Figure 4 shows the effective irradiance of UVR emitted during GTAW, SMAW, and GMAW of cast iron. The effective irradiances measured in this study were in the range of 0.045‐2.2 mW/cm^2^ at a distance of 500 mm from the welding arc. The maximum allowable exposure times corresponding to these levels were 1.4‐67 s/day.

The effective irradiance of UVR emitted during arc welding of cast iron measured under the welding conditions of this study, as well as other studies,^12^, ^13^, ^14^, ^15^, ^16^, ^17^ all increased with the increase in the welding current. Additionally, the effective irradiance of UVR emitted during arc welding of cast iron was different depending on the welding processes and was higher in the order of GMAW > SMAW > GTAW. The effective irradiance of UVR emitted in SMAW and GMAW was also confirmed to be affected by the components of the filler material used. The effective irradiance of SMAW was highest when using E4916, followed by ENi‐CI and ENiFe‐CI, but there was no significant difference between the latter two.

The effective irradiance of GMAW was higher with YS308 than with YGW12.

**Figure 5:**
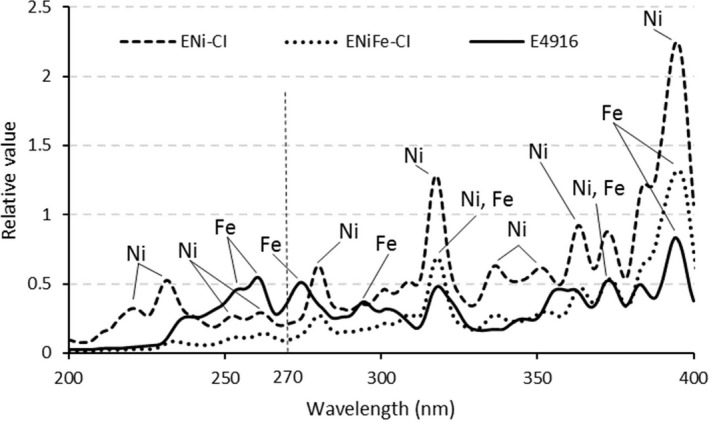
Spectral irradiance of UVR emitted by SMAW of cast iron using different welding rods. The welding current is 150 A. SMAW, shielded metal arc welding; UVR, ultraviolet radiation

Figure 5 shows the spectral irradiance of UVR emitted during SMAW of cast iron. Under all conditions, emission was observed from the component contained in the core wire of the covered electrode. When E4916 was used, emission from Fe was observed, and when using ENi‐CI, emission from Ni was observed. In addition, when ENiFe‐CI was used, emission from both Fe and Ni was observed. No clear emission from the coating flux component was observed under any condition.

**Figure 6:**
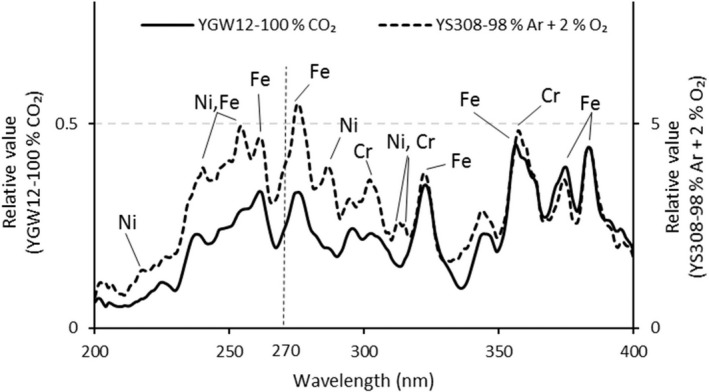
Spectral irradiance of UVR emitted by GMAW of cast iron using different welding wires. The welding current is 150 A. GMAW, gas metal arc welding; UVR, ultraviolet radiation

Figure 6 shows the spectral irradiance of UVR emitted during GMAW of cast iron. Under both conditions, emission from Fe, which is the main component of the wire, was observed. When YS308 was used, emission from Ni and Cr contained in the wire was observed. No clear emission was observed due to the shielding gas components, Ar and CO_2_, under any of the conditions.

## 4 DISCUSSION

Effective irradiances observed at distances of 500 mm from the arc were in the range 0.045‐2.2 mW/cm^2^. At these irradiances, the allowable daily exposure times are just 1.4‐67 seconds, which are extremely small compared to the cumulative exposure time over the course of a single day, indicating that direct exposure to UVR emitted during arc welding of cast iron is quite hazardous. Therefore, if workers engage in arc welding of cast iron without taking appropriate protective measures, even short‐time welding will result in exposure to dangerous amounts of UVR.

It is thought that workers are often exposed to UVR when striking their arcs. Generally, workers wear face shields^28^ equipped with filter plates^29^ as a countermeasure against UVR exposure when welding. However, since those filter plates have low visible light transmittance levels, it is difficult for workers to see the target welding point before the arc is generated, which means that the workers cannot don their face shields until just before the arcs are produced. As a result, workers are thought to be exposed to UVR because they often accidentally strike arcs before donning their face shields. Although the exposure for each arc strike is brief, the cumulative exposure is significant since workers usually strike an arc many times a day. Hence, the total exposure time might easily exceed the daily allowable exposure times obtained in this study. Therefore, every worker should always don a face shield before starting the arc. These days, the brief exposure to UVR that occurs when striking the arc can be easily avoided using auto‐darkening welding helmets, which are becoming increasingly mainstream in welding workplaces. Such auto‐darkening welding helmets are equipped with filters that can change transmittance levels and a sensor that detects light from the arc. The helmet filter darkens when the arc is produced. Therefore, unlike conventional face shields, an automatic dark welding helmet can be worn regardless of the presence or absence of the arc, which means that exposure to UVR can be avoided when the arc occurs.

If we assume that the effective irradiance of UVR decreases with the distance from the arc according to the inverse‐square law, the daily allowable exposure times at a distance of 5 m from the arc fall in the range of 1400‐6700 seconds. Thus, even at a distance of 5 m from the arc, exposure to UVR is hazardous in cases in which the emitted UVR is intense. Moreover, even in cases where the emitted UVR is weak, prolonged exposure is still hazardous.

In workplaces where arc welding is performed, other workers are often performing work unrelated to arc welding and it is thought that such workers can also be exposed to the UVR emitted during arc welding. Therefore, when performing other work in areas where welding is being performed, workers should wear personal optical radiation eye protectors that meet JIS,^29^ and workers should avoid exposing their skin to the arc. Furthermore, supervisors need to take measures, such as partitioning the location where arc welding is performed with a welding curtain, to prevent the nearby workers from being exposed to the UVR emitted by the arc.

Furthermore, as noted in other studies,^12^, ^13^, ^14^, ^15^, ^16^, ^17^ the effective irradiance of UVR emitted during GTAW, SMAW, and GMAW of cast iron increased with the increase in welding current. Therefore, it can be said that welding current is an important factor affecting the hazard of UVR emitted during arc welding of cast iron, as well as the arc welding of other metallic materials. It should also be noted that the effective irradiance of UVR emitted during arc welding of cast iron differs depending on the welding process, and its value was found to be higher in the order of GMAW > SMAW > GTAW.

Sliney et al^12^ measured the effective irradiance of UVR emitted by GTAW, SMAW, and GMAW of mild steel and found that the magnitude of effective irradiance emitted by each welding process increases in the order of GMAW > SMAW > GTAW. Since this tendency is consistent with the results of this study, it can be concluded that the welding process is also an important factor affecting the hazard of UVR emitted during the arc welding of cast iron.

The reason why the effective irradiance of UVR emitted by each welding processes is different is thought to be due to difference in the amount of metal vapor mixed into the arc column.^30^ In the case of GTAW, the supply source of metal vapor is just the molten pool of base metal because the electrode type used in this welding process is non‐consumable. In contrast, since SMAW and GMAW are consumable electrode type welding processes, not only the molten base metal pool but also the electrode filler material that has melted into the arc column can be a supply source of metal vapor. Therefore, since the amount of metal vapor generated in SMAW and GMAW is larger than that generated in GTAW, the effective irradiance of UVR emitted by SMAW and GMAW is considered to have increased.^16^, ^17^

The difference between GMAW and SMAW (GMAW > SMAW) is thought to be due to the difference in the current density due to the diameter of the filler material. The diameter of the welding wire used in GMAW is 1.2 mm. Furthermore, the diameter of the covered electrode used in SMAW is between 3.2 and 5.0 mm, and the current density of GMAW is about 10‐20 times that of SMAW. Since the current density is higher, the resistance heat generated in the filler material increases, and the melting rate of the filler material also increases.^31^ Therefore, it is considered likely that the effective irradiance of GMAW becomes higher than that of SMAW because more metal vapor exists in the arc column during GMAW.

The effective irradiance of UVR emitted during SMAW of cast iron was the highest when using E4916, and the levels were almost equal when using ENi‐CI and ENiFe‐CI. The difference in effective irradiance between E4916 and Ni‐type covered electrodes (ENi‐CI and ENiFe‐CI) is thought to be due to the difference in arc temperature caused by the two coating flux components. Usually, the coating flux of Ni‐type covered electrodes have the effect of lowering the welding voltage.^32^ For this reason, the arc temperature decreases. In contrast, the coating flux of E4916 tends to concentrate the arc,^11^ which causes the tip temperature of the covered electrode and the temperature of the arc column to increase, thereby resulting in more metal vapor entering the arc column, which might lead to a difference in effective irradiance.^17^

The spectral irradiance distribution of UVR emitted by SMAW shows the emission from Fe and Ni contained in the filler material (Figure 5). This indicates that the components contained in the filler material affect effective irradiance.

Focusing on the spectral irradiance distribution when E4916 is used, multiple strong light emissions from Fe were observed around the wavelengths of 240‐260 nm and at 275 nm (Figure 5). The relative spectral effectiveness^18^ was 0.30‐0.65 and 0.96 at these wavelengths (Figure 1). On the other hand, when ENi‐CI and ENiFe‐CI were used, clear emission from Ni was observed at a wavelength of approximately 280 nm. However, since the relative spectral effectiveness at this wavelength is 0.88, the effect of Ni on the effective irradiance was smaller than that of Fe. This could be the reason why the effective irradiance of E4916 was higher than that of Ni‐type covered electrodes. In addition, the spectral irradiance distribution with both types of Ni‐type covered electrodes showed almost the same distribution in the wavelength range (about 240‐300 nm) where the relative spectral effectiveness is large, suggesting no significant difference in the effective irradiance of the two.

The effective irradiance of UVR emitted during GMAW was highest in the case of YS308. When YS308 is used, the only luminescent element observed in the vicinity of the wavelength of 270 µm (where the relative spectral effectiveness is strongest) is Fe. In the past, Nakashima et al^15^, ^16^ measured the effective irradiance of UVR emitted during arc welding of aluminum alloys and found that the effective irradiance increases when Mg (alloy element) is contained in the base metal and filler material. This is due to the strong emission from Mg observed at wavelengths near 280 nm. In contrast, when YS308 was used in this study, light emission from Ni and Cr contained in the wire was observed in the wavelength range 290‐315 nm, but the effect on the effective irradiance was small. Therefore, it is considered likely that the effective irradiance of UVR emitted by GMAW measured in this study was strongly influenced by Fe, which is the main element of the welding wire.

The difference in effective irradiance between YS308 and YGW12 is considered to be the result of a difference in the amount of Fe vapor entering the arc column. The difference in the amount of Fe vapor is thought to be due to a difference in electrical resistance between the two wires. The electrical resistance of YS308 is more than four times higher than that of YGW12.^33^ As the resistance heat generated in the wire increases, the amount of wire melting increases as well.^31^ Therefore, it is possible that more metal vapor was present in the arc column with YS308.

## 5 CONCLUSION

The strong UVR emissions generated during arc welding of cast iron have the following characteristics:

(a) They are more hazardous at higher welding currents. (b) The magnitude of the hazard depends on the welding process, increasing in the order of GMAW > SMAW > GTAW. (c) The hazard level is affected by the filler material used, and more danger is present when E4916 is used in SMAW and YS308 is used in GMAW. (d) The components contained in the filler material affect the hazards of UVR, and the magnitude of the influence is Fe > Ni, Cr (e) The components (Ni, Cr) contained in the YS308 wire increase the electrical resistance of the wire, and the hazard of UVR is strengthened by the increasing amounts metal vapor entering the arc column.

## DISCLOSURE

*Approval of the research protocol*: N/A. *Informed consent*: N/A. *Registry and registration no. of the study/trial*: N/A. *Animal studies*: N/A. *Conflict of interest*: N/A.

## AUTHOR CONTRIBUTIONS

TO, NF, and HN conceived the ideas; TO, HN, and JT collected the data; HN and JT analyzed the data; NF, HN, and JT coordinated the writing.
